# Loss of ZG16 is associated with molecular and clinicopathological phenotypes of colorectal cancer

**DOI:** 10.1186/s12885-018-4337-2

**Published:** 2018-04-16

**Authors:** Hui Meng, Wencai Li, Lisa A. Boardman, Liang Wang

**Affiliations:** 1 Department of Pathology, First Affiliated Hospital of Zhengzhou University, Zhengzhou, Henan China; 2 Department of Pathology and MCW Cancer Center, Medical College of Wisconsin, Milwaukee, WI 53226 USA; 3 Department of Gastroenterology, Mayo Clinic, Rochester, MN 55905 USA

**Keywords:** Colorectal cancer, ZG16, Gene expression, Copy number variation, Survival

## Abstract

**Background:**

Zymogen granule protein 16 *(ZG16*) is one of the most significantly down-regulated genes in colorectal cancer (CRC) tissues. This study aimed to further evaluate its expression changes and investigate its association with molecular and clinicopathological characteristics of CRC.

**Methods:**

We applied quantitative RT-PCR to determine expression difference between tumor and matched normal tissues from 23 CRC patients. To further validate the down-regulation in tumor tissues, we performed immunohistochemistry (IHC) analysis in 40 paraffin-embedded normal-tumor pairs and 22 colon tissues with a variety of diseases. To evaluate if the *ZG16* gene changes were associated with clinicopathological characteristics, we further analyzed the gene expression and copy number changes from The Cancer Genome Atlas (TCGA) and Oncomine datasets.

**Results:**

Quantitative RT-PCR confirmed significant down-regulation (~ 130-fold) of *ZG16* in all tumor tissues. *ZG16* expression was in an organ-specific manner with an extremely high expression in normal epithelial cells of small intestine, colon and rectum. IHC analysis showed that *ZG16* protein was completely lost in all of 40 CRC tissues, and partially lost in premalignant adenomatous polyps (adenomas) and chronic ulcerative colitis tissues. Gene expression and copy number changes were significantly associated with multiple molecular and clinicopathological features of CRC including microsatellite instability (MSI), *MLH1* silencing, CpG island methylator phenotype, hyper-mutation status, gender, presence of synchronous adenomas, and histological type (*P* < 0.05). Patients with lower *ZG16* gene expression showed shorter progression-free survival and overall survival than those with relatively higher expression (*P* < 0.05). Multivariate analysis suggested that the *ZG16* expression was an independent prognosis factor (*P* = 0.012, HR = 6.286, 95% CI = 0.816–0.975).

**Conclusion:**

For the first time, our study demonstrated that *ZG16* expression was sequentially reduced from normal, adenoma, to carcinoma. Association with multiple clinicopathological features indicates that *ZG16* may play an important role in cancer initiation and progression. *ZG16* may serve as a potential biomarker for diagnosis and prognosis of CRC.

**Electronic supplementary material:**

The online version of this article (10.1186/s12885-018-4337-2) contains supplementary material, which is available to authorized users.

## Background

Colorectal cancer (CRC) is the third most common cancer and the third leading cause of cancer death in the US. Epidemiological studies show that high intake of some natural plants greatly reduce risk of CRC [1, 2]. This risk reduction is believed to be due to certain lectins, which include a group of proteins/glycoproteins of non-immune origin that bind specifically to carbohydrates. Lectins have the capacity to bind to specific complex carbohydrates based on subtle structural differences. Typically, lectins and their complementary carbohydrates are located on the surfaces of opposing cells, which can be of the same type or different types [3]. Their interactions are required for cell differentiation, development and pathological states. Cancer cells use carbohydrate moieties to escape recognition by the immune cells as they migrate through the body. During metastasis, the carbohydrates are involved in tumor cell–tumor cell, tumor cell–extracellular membrane or tumor cell–endothelial cell interactions. Pathogens rely on carbohydrates to home in on their tissue of choice in their preferred host species and to spread from cell to cell. Harmful inflammatory reactions are often triggered by carbohydrates, as is blood clotting. Many of the roles of cell surface carbohydrates and their binding proteins (lectins) in tumor angiogenesis and metastasis have been studied [4, 5]. For example, galectin (a well characterized lectin family) has been implicated in many essential functions including development, differentiation, cell–cell adhesion, cell–matrix interaction, growth regulation, apoptosis, RNA splicing, and tumor metastasis [6].

At least 12 structural lectin families are known to exist. Many lectins also bind structures other than carbohydrates via protein–protein, protein–lipid or protein–nucleic acid interactions. While lectins undoubtedly fulfil a variety of functions, many could be considered in general terms to be recognition molecules within the immune system. More specifically, lectins have been implicated in direct first-line defense against pathogens, cell trafficking, immune regulation and prevention of autoimmunity [7]. The lectin Jacalin is a glycoprotein extracted from Jackfruit (*Artocarpus heterophyllus*) seed [8]. It has a variety of important functions from T cell stimulation to tumor cell recognition and inhibition. While analyzing RNA expression profiles of CRC tissues, we found that zymogen granule protein 16 (*ZG16*) was one of the most significantly down-regulated genes among all RNA transcripts. Sequence analysis showed that the ZG16 protein contained a jacalin-like lectin domain. A previous study has shown that expression of this gene is restricted to normal epithelial cells of the small intestine, colon and rectum [9]. Using quantitative PCR and RNA in situ hybridization, Chen et al. reported down-regulation of *ZG16* in tumor tissues. When compared to patients without metastasis, patients with distant metastasis showed even more down-regulation [10]. Another recent study demonstrated that ZG16 may keep bacteria further away from the host colon epithelium [11], suggesting an important role of ZG16 in colon surveillance system.

To further explore the association of ZG16 with CRC development, we generated an anti-ZG16 polyclonal antibody and tested the gene expression in a panel of colon tissues with benign diseases including diverticulitis and ulcerative colitis, adenomas, and malignant diseases. We also analyzed *ZG16* copy number and gene expression in colon adenocarcinoma from The Cancer Genome Atlas (TCGA-COAD) and Oncomine dataset. We performed a correlative analysis between *ZG16* expression and molecular and clinicopathological characteristics of CRC patients. Our results from this study support an important role of *ZG16* in CRC initiation and progression.

## Methods

### Clinical specimens

Clinical samples were obtained at Mayo Clinic (Rochester, MN). For quantitative real-time PCR, we collected 23 pairs of snap-frozen normal/tumor colon tissues. For immunohistochemistry analysis, we evaluated 40 paraffin-embedded CRCs and their corresponding adjacent normal colon epithelium. Of the 40 pairs, 25 were adenocarcinoma and the remaining 15 were mucinous adenocarcinoma. We also collected tissues from a series of cancer-prone diseases including hamartomatous polyps from 2 Peutz-Jeghers syndrome (PJS) and 3 juvenile polyposis syndrome (JPS) patients; sporadic polyps from 10 patients without synchronous or metachronous CRC (tubular adenoma [TA] with low grade dysplasia [LGD] and 6 tubulovillous adenoma [TVA] with LGD), and affected colonic epithelium from 6 patients with chronic ulcerative colitis (CUC) and 1 with acute colitis. Dysplasia grading was based on 6th edition of the UICC and AJCC publications on the digestive tract.

### RNA extraction

Frozen tumor blocks were manually dissected to remove muscle and stroma and to enrich for cellular areas containing ≥ 70% cancer cells and then cut into 10 μm sections. Normal colon was dissected from an area at least 8 cm away from the tumor, manually dissected to enrich for mucosa, and then cut into 10 μm sections. All slides were reviewed by a pathologist to outline areas of interest. Frozen sections were placed into RNeasy RLT buffer (Qiagen, Valencia, CA) and stored at − 80 °C. After completing the RNA extraction, RNA was quantitated by nanodrop (Thermo Scientific, Wilmington, DE).

### Quantification of gene expression

The *ZG16* gene was analyzed by quantitative RT-PCR in a total of 23 normal/cancer pairs. Extracted RNA was transcribed into cDNA by SuperScript III reverse transcriptase according to manufacturer’s protocol (Life Technologies, Carlsbad, CA). All PCR reactions were performed using a LightCycler instrument (Roche Diagnostics, Indianapolis, IN). For each PCR run, a master mix was prepared on ice, including 10 pmol of each primer, 3-4 mM of MgCl2, and 1xSYBR Green I solution (Roche Diagnostics, Indianapolis, IN). 2ul of each RT product was added to 18ul of the PCR master-mix. The thermal cycling conditions comprised an initial denaturation step at 95 °C for 30s, 40 cycles at 95 °C for 5 s, and 58 °C for 5 s, and 72 °C for 10s. PCR primers for the gene is *forward-CCGAACAGTAGAGGCCTTCC, reverse -CGCACCTGAAGACCTACGAT*. As it was difficult to assess the precise amount of total RNA added to each reaction (based on absorbance) and its quality, we quantified a housekeeping gene glyceraldehyde-3-phosphate dehydrogenase (GAPDH) as the endogenous RNA control. The primers for the internal control were *forward-ACCACAGTCCATGCCATCAC* and *reverse-TCCACCACCCTGTTGCTGTA.* Each sample was run in duplicate and normalized to the internal control.

### Generation of antiserum polyclonal antibody

A more antigenic peptide corresponding to residues 22–46 of predicted protein (ARSSSYSGEYGSGGGKRFSHSGNQL) was synthesized at the scale of 5 mg. The peptide was used to immunize a rabbit and generate over 100 ml of antiserum by a standard immunization protocol (Rockland Inc., Limerick, PA). The polyclonal antibody from antiserum was purified through a peptide-bound affinity column (SulfoLink, Thermo Fisher Scientific Inc., Rockford, IL). This purified polyclonal antibody was used for western blot and immunohistochemistry (IHC) analysis.

### Western blot analysis

Whole cell lysates were prepared in Triton lysis buffer. Protein concentration was determined using a BCA protein assay kit (Thermo Fisher Scientific Inc.). Equal amount of cell lysates was separated on SDS-PAGE, and transferred to Hybond-P membranes. The membrane was blocked 1 h at room temperature using a blocking buffer and then incubated with anti-ZG16 antibody (1/500 dilution) overnight at 4 °C. After extensive washing, the membrane was incubated with the horseradish peroxidase-conjugated anti-rabbit immunoglobulin in blocking buffer at room temperature for 1 h. The membranes were washed three times in PBS prior to detection of bound antibody using the enhanced chemiluminescence (ECL) assay protocol (Thermo Fisher Scientific Inc.).

### Tissue microarray

Tissue microarrays were constructed from 0.6 mm cores of formalin-fixed paraffin-embedded human tissues. A normal tissue microarray was prepared from 20 different human organs. To ensure all tissues were free from any disease, each tissue was individually inspected by a pathologist. Tumor tissue microarrays consisted of 40 patient samples. For each patient, the array contained 10 colon tissue cores (6 from the cancer and 4 from the corresponding normal colon mucosa). Liver tissue cores were used as an internal control.

### IHC assay

The expression of the protein was assessed using IHC. Five μm tissue sections from formalin-fixed, paraffin-embedded tissues were deparaffinized through 3 changes of Xylene for 5 min each and rehydrated through graduated alcohols to running distilled water to rinse. The slides were then placed in a preheated 1 mM EDTA (pH 8.0) retrieval buffer for 30 min at which time they were cooled in the buffer for 5 min and placed in running distilled water for 5 min. The slides were then placed in the DAKO Cytomation Wash Buffer (10X w/Tween) for ~ 15 min. Primary antibody was diluted to 1/8000 and incubated for 30 min at room temperature. Staining was done on the DAKO Autostainer and visualized using the DAKO Cytomation EnVision Dual Link system. Upon completion of the staining, the slides were well rinsed in running distilled water for 5 min and then counterstained in a Modified Schmidts’ Hematox for 3 min, followed by a running tap water rinse for 3 min and then dehydrated to xylenes and mounted w/a Dako Permanent Mounting Media.

### TCGA colon cancer (COAD) dataset and statistical analysis

Detailed clinicopathological data for 463 primary colon cancer tissues and 86 normal colon tissues from TCGA colon cancer database were downloaded from UCSC Xena website (http://xena.ucsc.edu/). Among those, gene expression and DNA copy number profiles for *ZG16* were analyzed for 385 adenocarcinomas and 62 mucinous adenocarcinomas. Detailed phenotypes include colon polyps present, gender, lymphatic invasion, microsatellite instability, MLH1 silencing, hyper mutation status, CpG island methylated phenotype, treatment success status, histological type, sample type, recurrence free survival (RFS) in days, and overall survival (OS) in days. Student’s t-test was used to test statistical differences between different groups. Kaplan-Meier and Cox regression analyses were carried out to evaluate the associations between gene expressions and survival. A *P* value < 0.05 was considered significant. ONCOMINE dataset (www.oncomine.org/) was also examined to verify the association of *ZG16* expression and clinic-molecular changes including MLH1gene status, gender, treatment status, RFS and OS.

## Results

### Loss of *ZG16* in colon cancer tissues

**Fig. 1:**
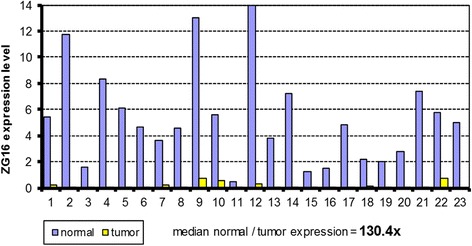
ZG16 expression in 23 normal-tumor pairs assayed by quantitative RT-PCR. Y-axis indicates normalized expression value in tumor-normal pairs (ΔCT value). ZG16 expression in tumor tissues is over 130-fold lower than in matched normal tissues

**Fig. 2:**
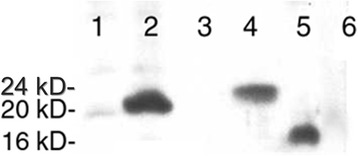
Western blot analysis of ZG16 protein under different conditions 1. Transfection-control; 2. Transfection- ZG16; 3. In vitro Translation-Negative control; 4. In vitro translation- ZG16; 5. Normal colon; and 6. Tumor colon

When analyzing expression profiles, *ZG16* was identified as one of the most significantly down-regulated genes in colon cancer datasets. To validate the gene expression changes, we performed quantitative RT-PCR in the 23 normal-tumor pairs and observed an average of ~ 130-fold decrease of RNA expression level in tumor tissues than in matched normal tissues (Fig. 1). To further analyze the gene expression at the protein level, we generated and purified an anti-ZG16 polyclonal antibody. We transfected a CHO cell line with pcDNA3.1 containing full length of ZG16 and a FLAG tag at C-terminal. We also performed in vitro translation using the same component vector. The products from these assays were subjected to western blot analysis along with a normal-tumor pair of colon tissues. The result showed that the antibody could specifically recognize 20kD, 24kD and 16kD proteins in the gene-specific transfection, in vitro translation and normal colon tissue, respectively. As expected, CRC tissue was negative (Fig. 2). Due to additional FLAG tag of transfection vector, natural protein in normal colon tissue was expected to be smaller than the proteins from gene transfection and in vitro translation. The different protein sizes between live CHO cells (20kD) and in vitro translation (24kD) indicated that the protein may be subjected to different posttranslational modifications in these different microenvironments.

**Fig. 3:**
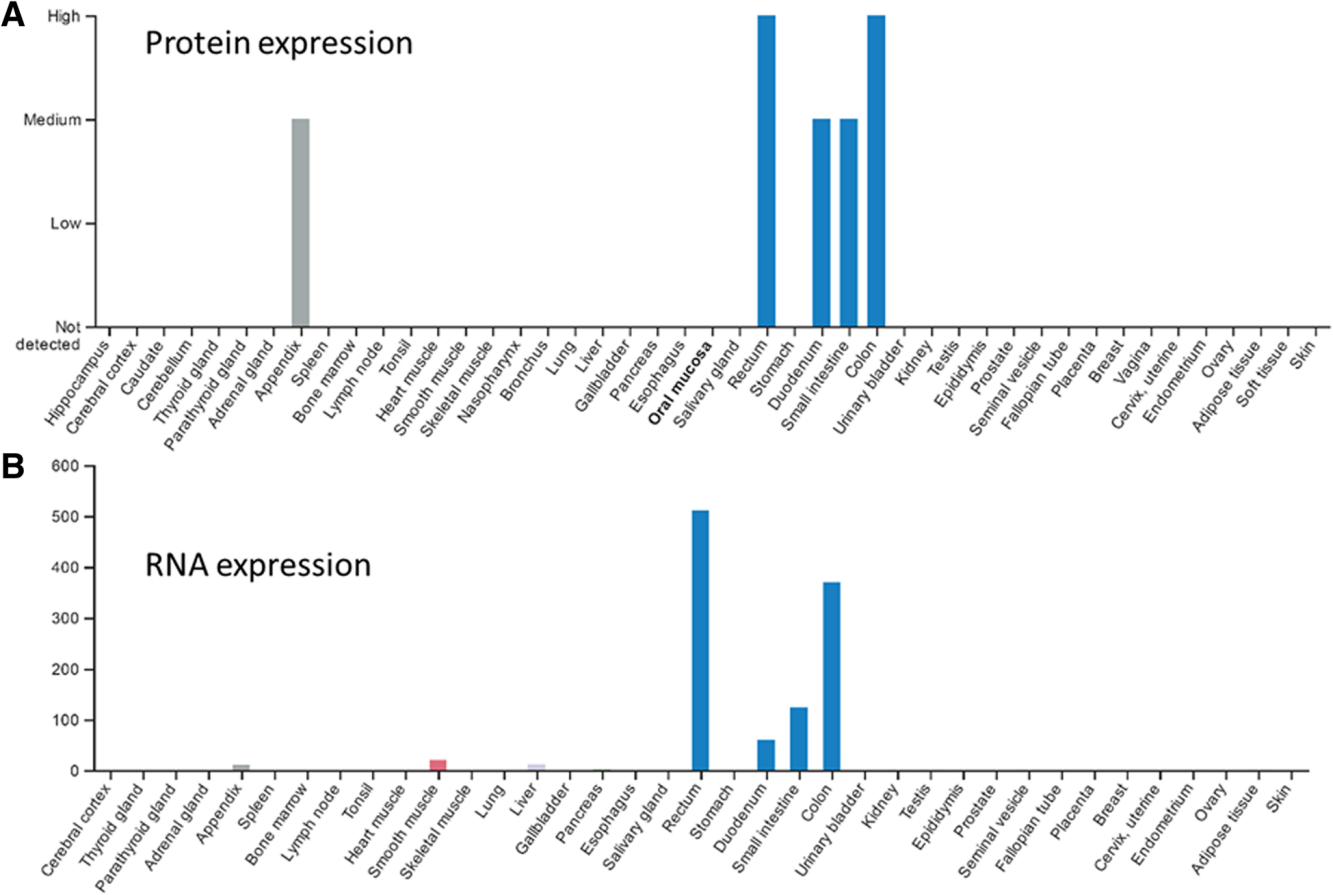
mRNA and protein expression in normal tissues collected from proteome project of Protein Atlas database. The highest ZG16 expression level is clearly seen in lower digestive tract system including duodenum, small intestine, colon and rectum at both protein (**a**) and RNA (**b**) levels

**Fig. 4:**
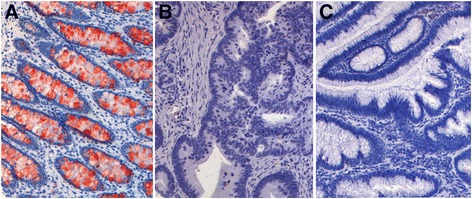
ZG16 expression in normal and tumor tissues by immunohistochemistry assay. **a** Normal colon tissue, **b** Adenocarcinoma, **c** Mucinous carcinoma. The protein is exclusively expressed in normal colon epithelial cells (**a**) while absent in tumors (**b** and **c**)

To examine overall expression of this protein in different human tissues, we checked the Protein Atlas database (http://www.proteinatlas.org). This proteome project collects gene and protein expression status in normal tissues, cancer tissues and different cell lines. This analysis revealed the highest expression level in lower digestive tract system including duodenum, small intestine, colon and rectum at both the protein and RNA level (Fig. 3). We also performed IHC analysis to test ZG16 expression in a multiple normal tissue microarray composed of 20 different human organs (Additional file 1: Table S1). This analysis confirmed the tissue-specific expression of ZG16 in colon tissues. To evaluate the protein expression in tumor tissues, we examined tissue array containing 25 cases of adenocarcinoma and their matched normal controls. Consistent with previous observation, the protein was exclusively expressed in normal colon epithelial cells while absent in tumors. The strongest expression was in the upper third of well-differentiated colon crypts. Since the expression was mainly located in goblet cells, we questioned whether the protein was a component of goblet cell-secreted mucins. We then collected and tested 15 paired mucinous colon tumors. None of these tumors actively expressed the protein (Fig. 4). We also examined the Oncomine database to explore the expression of ZG16 in normal and colon mucinous adenocarcinoma and adenocarcinoma tissues. We observed the same *ZG16* downregulation as in other samples (Additional file 2: Figure S1).

**Fig. 5:**
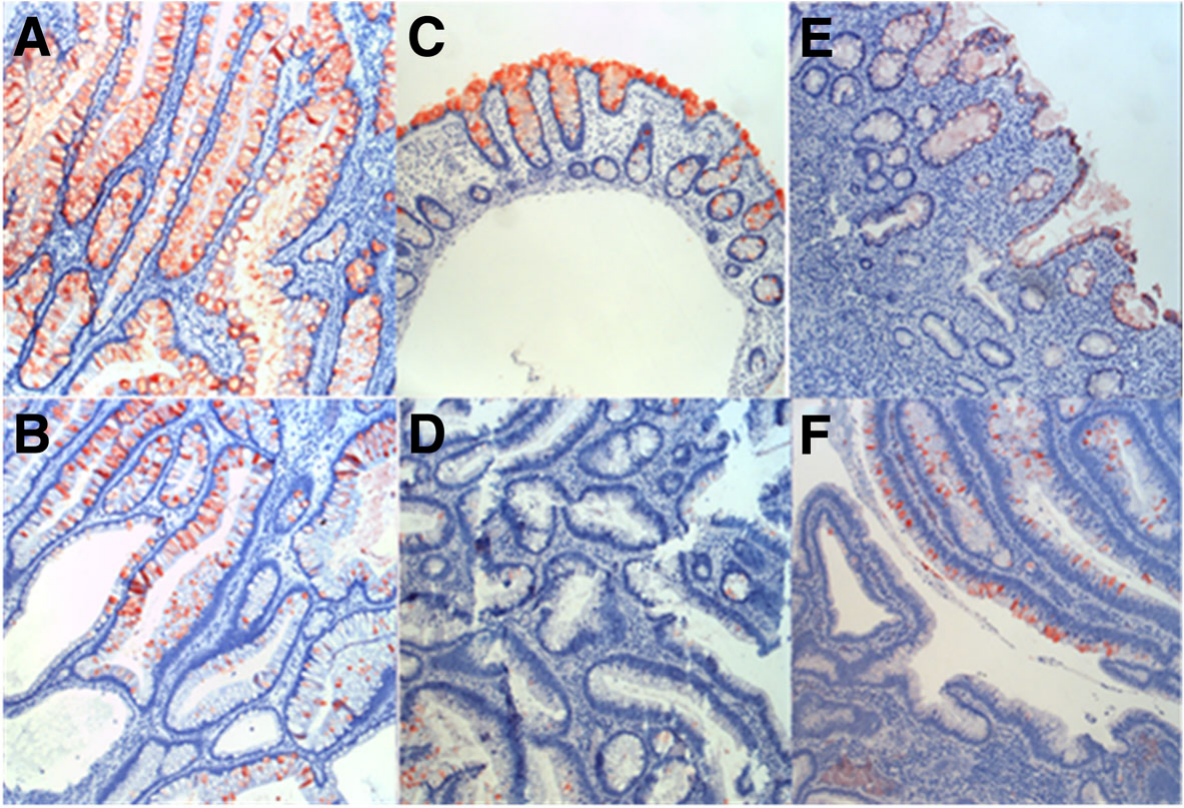
Expression of ZG16 in various diseases of colon. **a** Peutz-Jeghers syndrome, **b** Juvenile Polyposis syndrome, **c** Acute colitis, **d** Tubulous adenoma with low grade dysplaxia, **e** Chronic ulcerative colitis, **f** Tubulovillous adenoma with low grade dysplaxia

Because sporadic CRC are derived from sequential events (aberrant crypt foci-adenoma-carcinoma), we next performed IHC analysis in a series of cancer-prone diseases including 2 PJS, 3 JPS, 4 TA with LGD, 6 TVA with LGD, 6 CUC and 1 acute colitis. Except for 2 cases of PJS and 1 case of acute colitis, we observed clear reduction of the expression in all of these diseased tissues. The reduction ranged from focal (or local) expression in some areas to total loss in some other areas (Fig. 5, Table 1). The sequential reduction of *ZG16* expression was also observed from normal, adenoma, to carcinoma in Oncomine datasets (Additional file 2: Figure S2).

**Table 1:** Expression of ZG16 in normal and diseased colorectal epithelium

Diagnosis	Immunohistochemistry (IHC)	Positive cells %	Number of cases
Normal Colon	strong (+++)	95	*n* = 30
Polyps-PJS	strong (+++)	95	*n* = 2
Polyps-JPS	Positive (++) to negative	50	*n* = 3
TA with LGD	Positive (++) to negative	15	*n* = 4
TVA with LGD	Positive (+) to negative	10	*n* = 6
Acute Colitis	Positive (+++)	95	*n* = 1
CUC without colon cancer	Positive (++) to negative	20	*n* = 4
CUC with colon cancer	Positive (+) to negative	10	*n* = 2
Colorectal adenocarcinoma	negative	0	*n* = 25
Colorectal mucinous adenocarcinoma	negative	0	*n* = 15

*PJS*: Peutz-Jeghers syndrome, *JPS*: juvenile polyposis syndrome, *TA*: tubulous adenoma, *LGD*: low grade dysplaxia, *TVA*: tubulovillous adenoma, *CUC*: chronic ulcerative colitis

### *ZG16* gene expression changes and clinicopathological phenotypes

To evaluate clinical correlation of *ZG16* expression changes, we examined the gene expression using the TCGA-COAD data. When compared to male patients (*n* = 94, Table 2), female patients (*n* = 99) showed lower *ZG16* expression (*P* = 0.037). *ZG16* gene expression showed significantly higher in descending (*n* = 20) colon than ascending (*n* = 86) colon (*P* = 0.020). When comparing MSI status, MSI-high cancer tissues showed significantly lower expression than MSI-low tissues (*P* = 0.037). Correspondingly, the down-regulation was also observed in the CRC tissues with absence of *MLH1* (*P* = 0.043) and with evidence of hyper-mutation (*P* = 0.018). The ZG-16 expression also showed a significant association with MLH1 methylation status in another microarray dataset (Additional file 2: Figure S3). However, no association of the gene expression level with following phenotypes including colon polyps, histological type, lymphatic invasion, treatment success status, CpG island methylator phenotype was observed (*P* > 0.05). Table 2 lists detail statistics of the clinicopathological association.

**Table 2:** Correlations of ZG16 expression with clinicopathological features of colorectal cancer (TCGA COAD dataset)

Clinical-pathological features		Number of cases	Gene expression (average)	*P*
Gender				
	male	94	0.512	0.037
	female	99	− 0.487	
Colon polyps present				
	yes	73	−0.768	0.246
	no	129	−1.109	
Anatomic neoplasm subdivision				
	ascending colon	86	0.499	0.020
	descending colon	20	1.681	
Histological type				
	adenocarcinoma	62	−0.98	0.244
	mucinous- adenocarcinoma	385	−0.41	
Lymphatic invasion				
	yes	77	−1.136	0.793
	no	175	−1.032	
Pathological-N	N2 N0	48,167	− 1.464 -0.806	0.0072
Hyper-mutation status				
	true	32	−1.23	0.018
	false	119	0.571	
Microsatellite instability status				
	MSI-H	25	−1.58	0.037
	MSI-L	25	1.13	
	MSS	98	0.491	
MLH1 silencing				
	yes	20	−1.143	0.043
	no	120	0.563	
CpG island methylator phenotype				
	CIMP-H	29	−0.74	0.38
	CIMP-L	35	−0.24	
Treatment success status				
	complete remission	92	−0.49	0.080
	progressive	28	−1.91	
Sample type				
	primary tumor	286	−1.61	0.000
	tissue normal	46	14.59	

### Association of *ZG16* expression and survival

**Fig. 6:**
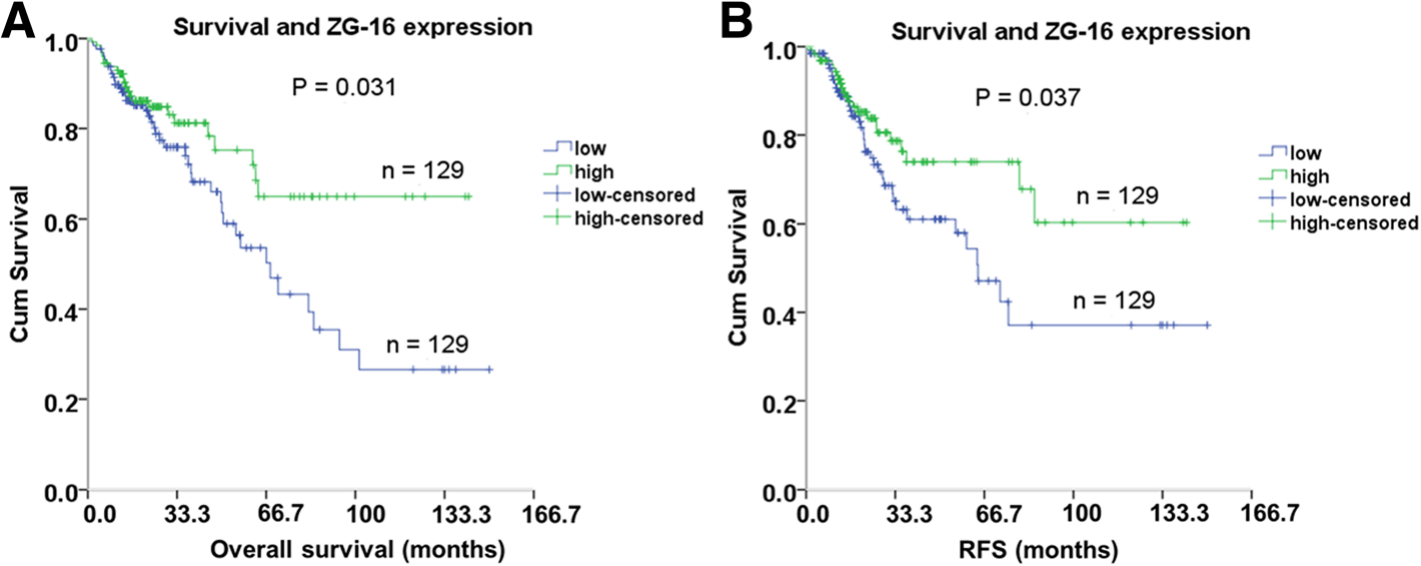
Association of *ZG16* expression and survival. Kaplan-Meier curve shows association of ZG16 with overall survival time (**a**, *P* = 0.031) and recurrence free survival time (**b**, *P* = 0.037) in 258 colon cancer patients

To test the association of *ZG16* gene expression with survival, we performed Kaplan-Meier analysis in 259 patients with OS and RFS data. Among the 259 patients, the median follow-up time was 22.8 months, ranging from 0 to 150 months. During the follow-up period, 181 patients died. This analysis showed that lower *ZG16* gene expression was significantly associated with poor survival (HR = 0.579; *p* = 0.031, Fig. 6a). The five- and ten-year survival rates were 72% and 65%, respectively, in the high expression group, and 53% and 26.6% in the low expression group. We also tested for the association of gene expression with RFS. Among 259 patients, 197 were free from recurrence during the follow up period. Kaplan-Meier analysis showed that lower *ZG16* expression was significantly associated with shorter RFS (HR = 0.563; *P* = 0.037, Fig. 6a). A subsequent multivariate analysis with OS showed that the gene expression was an independent prognosis factor (*p* = 0.012, HR = 6.286, 95% CI = 0.816–0.975, Table 3). The survival association was also seen in Oncomine databsets (Additional file 2: Figure S4). In one dataset, *ZG16* showed significantly higher expression in radiotherapy responders (*N* = 11) when compared to non-responders (*N* = 35) (*P* = 0.03) (Additional file 2: Figure S5). However, lower expression seemed associated with better response to modified FOLFOX.

**Table 3:** Multivariate Cox regression analysis of ZG16 expression as prognostic factors

Clinical-pathological features	P	HR	95% CI
Pathological N0/N2* Stages	0.000	3.274	1.798–5.962
Histological type 1/2^#^	0.046	2.142	1.015–4.519
ZG16 gene expression	0.012	0.892	0.816–0.975

*N0/N2 indicate lymphatic metastasis in TNM stages; *N0*: tumor cells absent from regional lymph nodes; *N2*: tumor spread to an extent between N1 and N3. ^*#*^*1*: adenocarcinoma; *2*: mucinous adenocarcinoma

### *ZG16* copy number changes and clinicopathological phenotypes

To evaluate if copy number variation (CNV) were associated with clinicopathological features, we performed detailed analysis using 447 patients with CNV data. Among those, 62 patients had mucinous adenocarcinoma while 385 patients had adenocarcinoma. When the two histological types were compared, *ZG16* copy number was significantly lower in tumors with mucinous adenocarcinoma than nonmucinous adenocarcinoma (*p* = 0.006). For tumor tissues with polyps present (*n* = 80), *ZG16* copy number level was significantly lower when compared to tumor tissues without polyps present (*n* = 148) (*P* = 0.031). We also found that tumors in the ascending colon (*n* = 86) had lower copy number than in descending colon (*n* = 20) (*p* = 0.028, Table 4).

**Table 4:** Significant correlations of ZG16 copy number and colon cancer (TCGA COAD dataset)

Clinical-pathological features		Number of cases	Copy number (Average)	*P*
Colon polyps present				
	yes	80	0.0309	0.031
	no	148	0.0778	
Anatomic neoplasm subdivision				
	ascending colon	86	0.108	0.028
	descending colon	20	0.378	
Histological type				
	adenocarcinoma	62	0.07	0.006
	mucinous- adenocarcinoma	385	−0.015	
Hyper-mutation status				
	TRUE	32	−0.012	0
	FALSE	121	0.0972	
Microsatellite instability status				
	MSI-H	25	−0.0234	0.003
	MSI-L	25	0.0657	
	MSS	100	0.101	
MLH1 silencing				
	yes	20	−0.0227	2.39E-09
	no	121	0.0952	
CpG island methylator phenotype				
	CIMP-H	31	0.011	0.01
	CIMP-L	32	0.084	
Treatment success status				
	complete remission	178	0.077	0.046
	progressive	36	0.172	

We also examined the association of copy number changes with MSI status. Among 150 cases, 25 cases were MSI-high, 25 cases were MSI-low and the remaining 100 cases were MSS (microsatellite stable). When compared to MSS and MSI-low tissues, MSI-high tissues showed significantly lower copy number at the *ZG16* locus (*p* = 0.003). The lower copy number was also observed in the cancers with *MLH*-silencing and hyper-mutation (*p* < = 2.39e-9). When comparing CpG island methylator phenotypes, CIMP-H cancer tissues had significantly lower copy number than CIMP-L cancer tissue (*p* = 0.01). Interestingly, patients with complete remission showed much lower copy number of *ZG16* than patients with progressive disease (*p* = 0.046). The detail statistical result is listed in Table 4.

### Bioinformatic prediction of ZG16 function

To predict potential function of *ZG16* gene, we first performed sequence similarity analysis. The gene has 3 exons encompassing about 1258 bp genomic region at 16p13.3 and the predicted protein consists of 167 amino acids. Mouse and rat homologues of the protein also have 167 amino acids. There was over 83% identity among the three homologues (Additional file 2: Figure S6A). Sequence alignment between ZG16 and Jackfruit Jacalin α chain showed over 28% identity and 58% similarity over 131 amino acids of ZG16 lectin domain (Additional file 2: Figure S6B). We next applied computer programs including InterPro at https://www.ebi.ac.uk/interpro/ and SignalP at http://www.cbs.dtu.dk/services/SignalP/ to further examine functional domain. Both programs revealed that the protein contains a signal peptide with 17 amino acids at its N-terminus and a jacalin-like lectin domain with 113 amino acids (Additional file 2: Figure S6A). We also applied computer programs including COMPARTMENTS at https://compartments.jensenlab.org and CELLO at http://cello.life.nctu.edu.tw/cello2go/ to predict subcellular localization. The results from these programs demonstrated that ZG16 protein was more likely localized at Golgi lumen and extracellular matrix (Additional file 2: Figure S7).

## Discussion

In this study, we quantified *ZG16* expression at mRNA level by RT-PCR and at protein level by IHC in a large panel of CRC tissues. Our results further confirmed significant down-regulation of *ZG16* gene expression in CRC. This down-regulation is associated with clinicopathological and molecular characteristics of these patients. CRC rarely, if ever, arises de novo from normal mucosa. Rather, all cancers appear to arise from a nonmalignant but dysplastic adenoma. Through systematic evaluation by IHC, our data showed sequential down-regulation of *ZG16* from partial loss of its expression in the neoplastic precursor adenomatous polyps to complete loss in the resultant adenocarcinoma, support the role of ZG16 loss as early event during CRC initiation [12].

Early detection is important to reduce CRC mortality. Current screening tests include fecal occult blood testing, stool based DNA testing [12], computed tomography imaging, and colonoscopy [13]. Additionally, molecular analysis of colonoscopy-based biopsied tissues has proven invaluable for CRC prognostication [14]. Our data showed that sequential down-regulation of *ZG16* from early precancerous lesions to adenocarcinoma was associated with the molecular phenotypes of these tumors being MSI-H and CIMP-H, and/or exhibiting MLH1 silencing. These biomarkers are commonly used to examine both premalignant adenomas and CRC to determine molecular pathway alterations [14]. Additionally, lower *ZG16* expression conferred poor OS, which is consistent with a recent small study [10]. Furthermore, when compared to adenocarcinoma, patients with mucinous adenocarcinoma of colon (a more aggressive subtype) had lower copy number of *ZG16*. Thus, *ZG16* might serve as a sensitive molecular marker for early detection and outcome prediction of colon cancer [15].

Protein sequence analysis showed that ZG16 contains a signal peptide, suggesting that it is a secretory protein upon maturation. Direct secretion of the protein into digestive track implicates its potential role in colon surveillance. In fact, a recent study examined the role of ZG16 in protecting bacterial penetration into mucosa layer by comparing Zg16^−/−^ to Zg16^+/+^ mice [11]. This study demonstrated that ZG16 can aggregate Gram-positive bacteria and prevent bacterial penetration into the mucus. The study also demonstrated an increased 16S caudal lymph node load and viable splenic bacteria, as well as higher levels of serum proinflammatory cytokines in Zg16^−/−^ mice. Therefore, ZG16 may be an essential component of the mucus barrier and its presence may maintain bacteria at a safe distance from epithelial cell surface. Absence of the mucus layer may allow bacteria to come in close contact with the epithelium and cause an increased bacterial uptake and local inflammation [16, 17].

Interestingly, ZG16 protein has a jacalin-like lectin domain, which is a major part of the mature protein, further implicating its role in colon immune recognition. Jacalin is a two-chain lectin consisting of an α chain of 133 amino acid residues noncovalently bound to β chain of 20 or 21 amino acid residues [18, 19]. High similarity between ZG16 and jacalin α chain supports ZG16 as a human homologue of Jackfruit Jacalin. Study shows that Jacalin displays strong affinity for Galβ1–3GalNAc, the Thomsen–Friedenreich (TF) antigen so as to perform function of recognition, differentiation and anti-proliferation of tumor cells. Since the TF-antigen is an important marker in human carcinoma, the possibility that Jacalin can be used as an anti-TF antigen has been suggested [20, 21]. Jacalin also selectively binds to MUC1, an oncoprotein. Mucins have been implicated in tumor-associated immunosuppression [22]. Soluble colon cancer mucins containing mucins MUC1/2 inhibited IL-2 mRNA expression and secretion of CD4+ [23]. The high similarity of ZG16 to Jacalin indicates that the human homologue may play an important role in colon cancer immunity. Based on these results, we propose that by limiting bacterial translocation into the host, ZG16 may not only prevent local inflammation but also play an important role in colon tumorigenesis.

## Conclusion

Primarily found in mature epithelial cells of lower digestive system, ZG16 may be an important component of protective mucus layer, which helps separate host epithelium from commensal bacteria in colon. For the first time, we showed sequential down-regulation of ZG16 during CRC progression and identified significant association of ZG16 with molecular and clinicopathological phenotypes of CRC. Due to its significant loss in CRC tissues and high similarity with jacalin chain, we hypothesize that ZG16 loss may disrupt the well-organized surveillance system, facilitate bacteria invasion into host system and cause local inflammatory changes, which may be directly associated with an increased risk to cancer development. To confirm this hypothesis, more in vitro and in vivo experiments are needed including microbiota analysis and conditional knockout mouse for carcinogenesis study.

## Additional files

Additional file 1: **Table S1.** Immunohistochemistry analysis of ZG-6 expression in different human tissues. (DOCX 13 kb)

Additional file 2: Additional ZG16 expression profiles, protein similarity and subcellular localization. (DOCX 794 kb)

## Abbreviations

CIMP: CpG island methylator phenotype
CNV: Copy number variation
CRC: Colorectal cancer
IHC: Immunohistochemistry
MSI: Microsatellite instability
OS: Overall survival
RFS: Recurrence-free survival
TCGA: The cancer genome atlas
TF: Thomsen–Friedenreich
ZG16: Zymogen granule protein 16
