# Supplementary material for: Loss of ZG16 is associated with molecular and clinicopathological phenotypes of colorectal cancer
Source: BMC Cancer. 2018 Apr 16;18:433. doi: 10.1186/s12885-018-4337-2 (PMC5902988; doi:10.1186/s12885-018-4337-2)
Supplement: Supplementary file 1 — Table S1. Immunohistochemistry analysis of ZG-6 expression in different human tissues. (DOCX 13 kb) [file 12885_2018_4337_MOESM1_ESM.docx]

| **Additional file 1: Table S1. Immunohistochemistry analysis of ZG-6 expression in different human tissues** | | | | | | | | | |
| --- | --- | --- | --- | --- | --- | --- | --- | --- | --- |
| Esophagus IS (negative) | Esophagus XS (negative) | Stomach mucosa IS (negative) | Stomach muscularis (negative) | Liver  (weak+) | Gall bladder (negative) | Small bowel mucosa (strong+++++) | Sigmoid colon mucosa (strong+++++) | Sigmoid colon muscularis (negative) | Colon submucosa (strong+++++) |
| Kidney glomerula (weak+) | Kidney tubules (weak+) | Bladder mucosa (negative) | Bladder wall (negative) | Liver  (weak+) | Ureter (negative) | Pancreas (negative) | Adrenal (weak+) | Thyroid (negative) | Tonsil (negative) |
| Lymph node (negative) | Spleen (negative) | Thymus, preinvoluted (negative) | Thymus involuted (negative) | Liver  (weak+) | Skeletal muscle (negative) | Lung bronchioles (negative) | Lung focal (weak+) | Heart epicardium (negative) | Heart myocardium (negative) |
| Hippocampus nuclus (weak+) | Hippocampus nuclus (weak+) | Skin (thin) (negative) | Hypodemis (negative) | Liver  (weak+) | Breast ducts (negative) | Breast stoma (negative) | Ovary (negative) | Uterus (negative) | Fallopian tube (negative) |
|  |  |  |  |  |  |  |  |  |  |
| Cervix (negative) | Plancenta (negative) | Testis (weak+) | Prostate (negative) | Liver  (weak+) | Gall bladder (negative) | Kidney glomerula (focal weak+) | Adrenal (negative) | Tonsil (negative) | Spleen (negative) |
|  |  |  |  |  |  |  |  |  |  |
| Liver  (weak+) | Liver  (weak+) | Thymus, preinvoluted (negative) | Skeletal muscle (negative) | Liver  (weak+) | Lung (negative) | Heart myocardium (negative) | Skin (thin) (negative) | Breast ducts (negative) | Prostate (weak+) |
