# Supplementary material for: Loss of ZG16 is associated with molecular and clinicopathological phenotypes of colorectal cancer
Source: BMC Cancer. 2018 Apr 16;18:433. doi: 10.1186/s12885-018-4337-2 (PMC5902988; doi:10.1186/s12885-018-4337-2)
Supplement: Supplementary file 2 — Additional ZG16 expression profiles, protein similarity and subcellular localization. (DOCX 794 kb) [file 12885_2018_4337_MOESM2_ESM.docx]

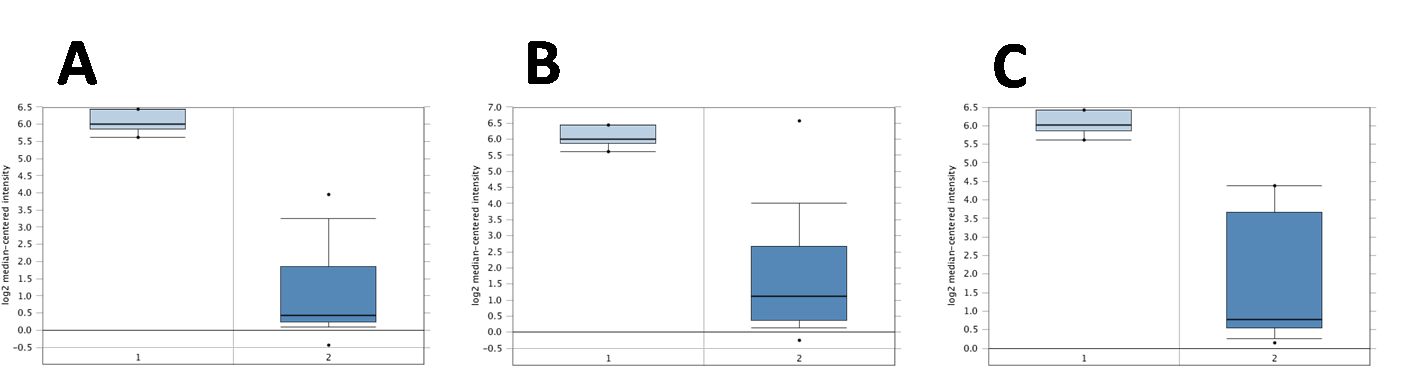


**Additional file 2: Figure S1. Downregulation of *ZG16* expression in colon cancer (Oncomine).** We examined Oncomine datasets and observed consistent down-regulation of ZG16 in tumor tissues than matched normal tissues. The figure shows one (*Kaiser et al,Genome Biol. 2007;8:131*) of many datasets with the down-regulation. A1, B1 and C1: Normal (N=5). A2: Cecum adenocarcinoma (N=17): B2: Colon mucinous adenocarcinoma (N=41). C2: Rectosigmoid adenocarcinoma (N=10). *P<*1.73E-6.

Kaiser S, Park YK, Franklin JL, Halberg RB, Yu M, Jessen WJ, Freudenberg J, Chen X, Haigis K, Jegga AG, et al. Transcriptional recapitulation and subversion of embryonic colon development by mouse colon tumor models and human colon cancer. Genome Biol. 2007;8:131.


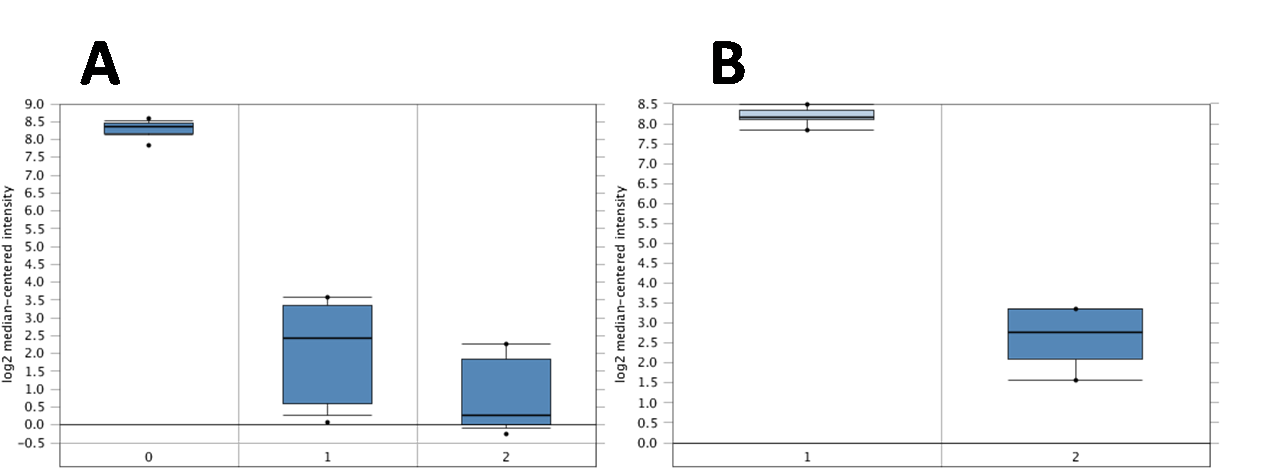


**Additional file 2: Figure S2. Sequential downregulation of *ZG-16* during disease progression (Oncomine).** Grouped by cancer type. A0: Colon (N=20), A1: Colon adenoma (N=10, *Skrzypczak et al, 2010;5:10*). A2: Colon carcinoma (N=10). B1: Colon (N=10). B2: Colon adenoma (N=5, *Gaspar C1et al, 2008;172:1363-80*).

[Skrzypczak M](https://www.ncbi.nlm.nih.gov/pubmed/?term=Skrzypczak%20M%5BAuthor%5D&cauthor=true&cauthor_uid=20957034), [Goryca K](https://www.ncbi.nlm.nih.gov/pubmed/?term=Goryca%20K%5BAuthor%5D&cauthor=true&cauthor_uid=20957034), [Rubel T](https://www.ncbi.nlm.nih.gov/pubmed/?term=Rubel%20T%5BAuthor%5D&cauthor=true&cauthor_uid=20957034), [Paziewska A](https://www.ncbi.nlm.nih.gov/pubmed/?term=Paziewska%20A%5BAuthor%5D&cauthor=true&cauthor_uid=20957034), [Mikula M](https://www.ncbi.nlm.nih.gov/pubmed/?term=Mikula%20M%5BAuthor%5D&cauthor=true&cauthor_uid=20957034), [Jarosz D](https://www.ncbi.nlm.nih.gov/pubmed/?term=Jarosz%20D%5BAuthor%5D&cauthor=true&cauthor_uid=20957034), [Pachlewski J](https://www.ncbi.nlm.nih.gov/pubmed/?term=Pachlewski%20J%5BAuthor%5D&cauthor=true&cauthor_uid=20957034), [Oledzki J](https://www.ncbi.nlm.nih.gov/pubmed/?term=Oledzki%20J%5BAuthor%5D&cauthor=true&cauthor_uid=20957034), [Ostrowski J](https://www.ncbi.nlm.nih.gov/pubmed/?term=Ostrowski%20J%5BAuthor%5D&cauthor=true&cauthor_uid=20957034). Modeling oncogenic signaling in colon tumors by multidirectional analyses of microarray data directed for maximization of analytical reliability. PLoS One. 2010;5:10.

Gaspar C, Cardoso J, Franken P, Molenaar L, Morreau H, Möslein G, Sampson J, Boer JM, de Menezes RX, Fodde R.Cross-species comparison of human and mouse intestinal polyps reveals conserved mechanisms in adenomatous polyposis coli (APC)-driven tumorigenesis. Am J Pathol. 2008;172:1363-80.


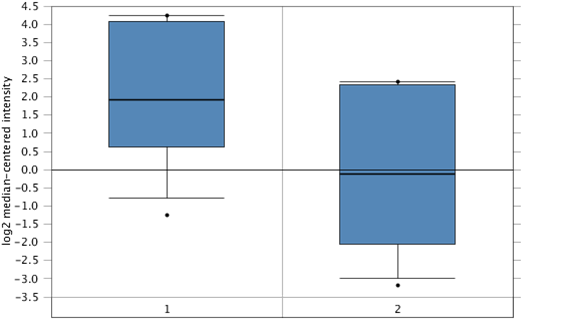


**Additional file 2: Figure S3. Association of *ZG16* expression with *MLH1* methylation status (Oncomine).** 1: *MLH1*methylation negative (N=10). 2: *MLH1* methylation positive (N=10).

Koinuma K, Yamashita Y, Liu W, Hatanaka H, Kurashina K, Wada T, Takada S, Kaneda R, Choi YL, Fujiwara SI, Miyakura Y, Nagai H, Mano H. Epigenetic silencing of AXIN2 in colorectal carcinoma with microsatellite instability. Oncogene. 2006;1:139-46.


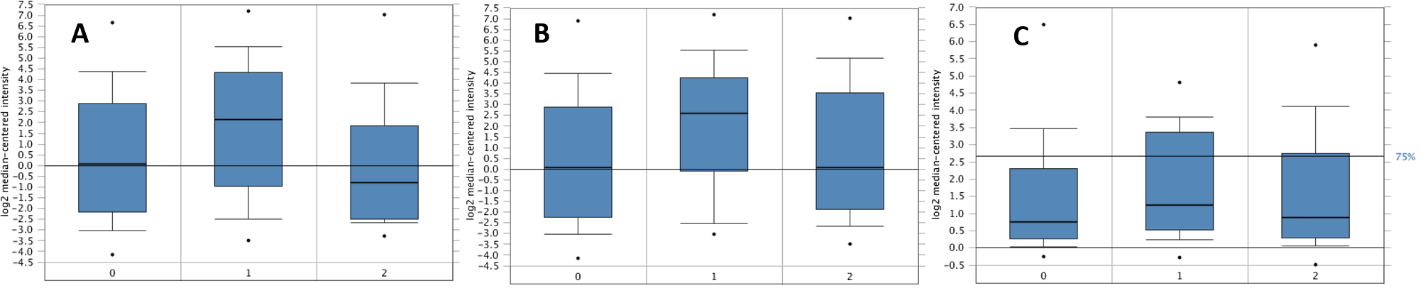


**Additional file 2: Figure S4. Association of ZG16 expression with recurrence and survival (Oncomine).** Higher expression is associated with progression-free survival (no recurrence) and overall survival. A0: No value (N=55). A1: No recurrence at 3 Years (N=79). A2: Recurrence at 3 years (N=20) (*Staub E et al, J Mol Med (Berl). 2009;87:633-44*). B0: No value (N=59). B1: No recurrence at 5 Years (N=37). B2: Recurrence at 5 years (N=58) (*Lin YH et al, Clin Cancer Res. 2007;13:498-507*). C0: No value (N=58). C1: Alive at 5 years (*N=52*). C2: Dead at 5 years (N=67) (*Staub E et al, J Mol Med (Berl).2009;87:633-44)*.

Staub E, Groene J, Heinze M, Mennerich D, Roepcke S, Klaman I, Hinzmann B, Castanos-Velez E, Pilarsky C, Mann B, Brümmendorf T, Weber B, Buhr HJ, Rosenthal A. An expression module of WIPF1-coexpressed genes identifies patients with favorable prognosis in three tumor types. J Mol Med (Berl). 2009;87:633-44.

Lin YH, Friederichs J, Black MA, Mages J, Rosenberg R, Guilford PJ, Phillips V, Thompson-Fawcett M, Kasabov N, Toro T, Merrie AE, van Rij A, Yoon HS, McCall JL, Siewert JR, Holzmann B, Reeve AE.Multiple gene expression classifiers from different array platforms predict poor prognosis of colorectal cancer.Clin Cancer Res. 2007;13:498-507.


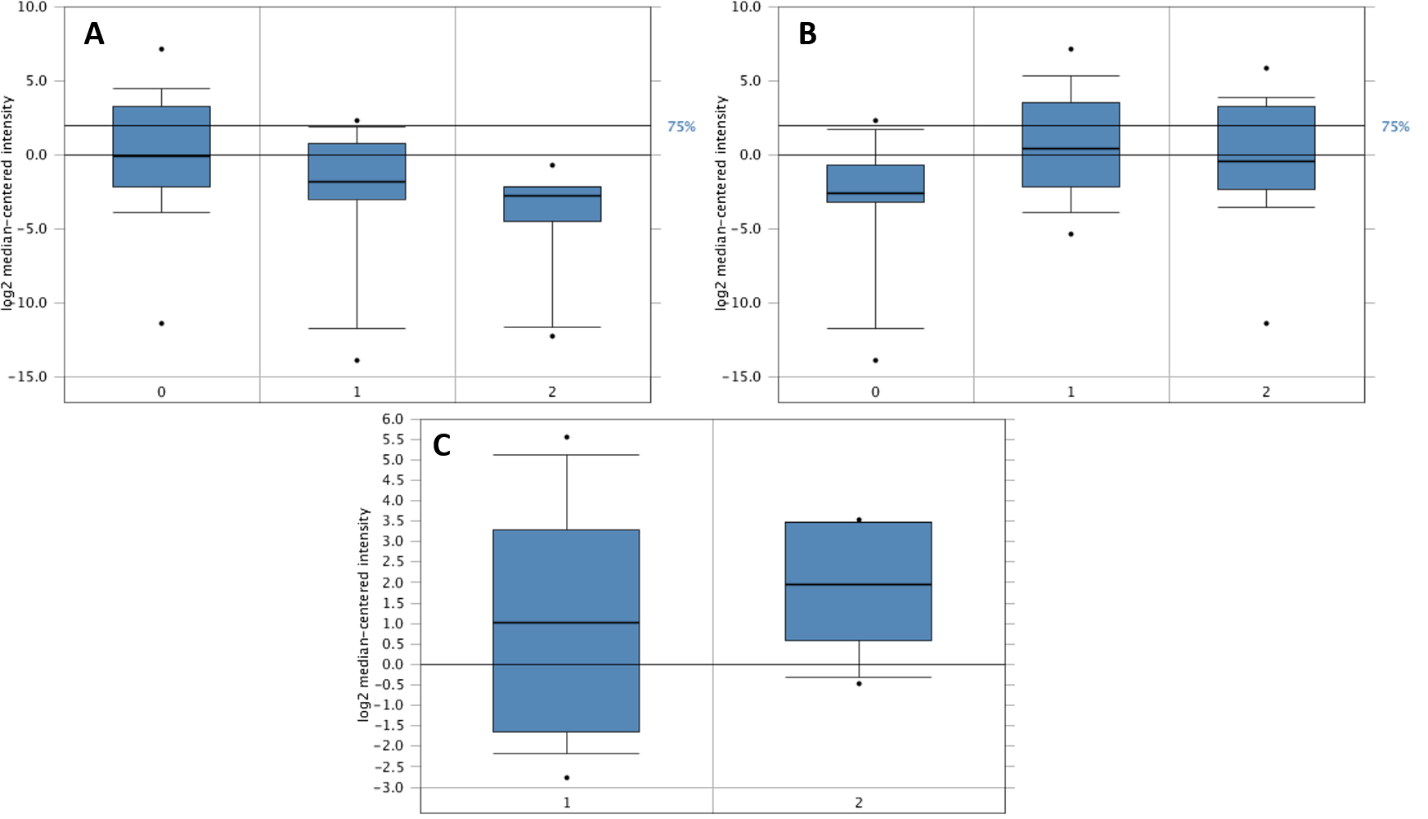


**Additional file 2: Figure S5. Association of *ZG16* expression with treatment responses (Oncomine).** A. Response to modified FOLFOX 6 (metastaticcolorectal carcinoma) (*Tsuji et al, Br J Cancer. 2012;106:126-32*). A0: No value (N=56). A1: Non-responder (N=16). A2: Responder (N=11). B. Response to modified FOLFOX 6. B0: No value (N=27). B1: Non-responder (N=25). B2: Responder (N=31). C. Response to radiotherapy (*Watanabe et al, Cancer Res. 2006;66:3370-4*). C1: Non-responder (N=35), C2: Responder (N=11).

Tsuji S, Midorikawa Y, Takahashi T, Yagi K, Takayama T, Yoshida K, Sugiyama Y, Aburatani H. Potential responders to FOLFOX therapy for colorectal cancer by Random Forests analysis. Br J Cancer. 2012;106:126-32.

Watanabe T, Komuro Y, Kiyomatsu T, Kanazawa T, Kazama Y, Tanaka J, Tanaka T, Yamamoto Y, Shirane M, Muto T, Nagawa H.Prediction of sensitivity of rectal cancer cells in response to preoperative radiotherapy by DNA microarray analysis of gene expression profiles. Cancer Res. 2006; 66:3370-4.


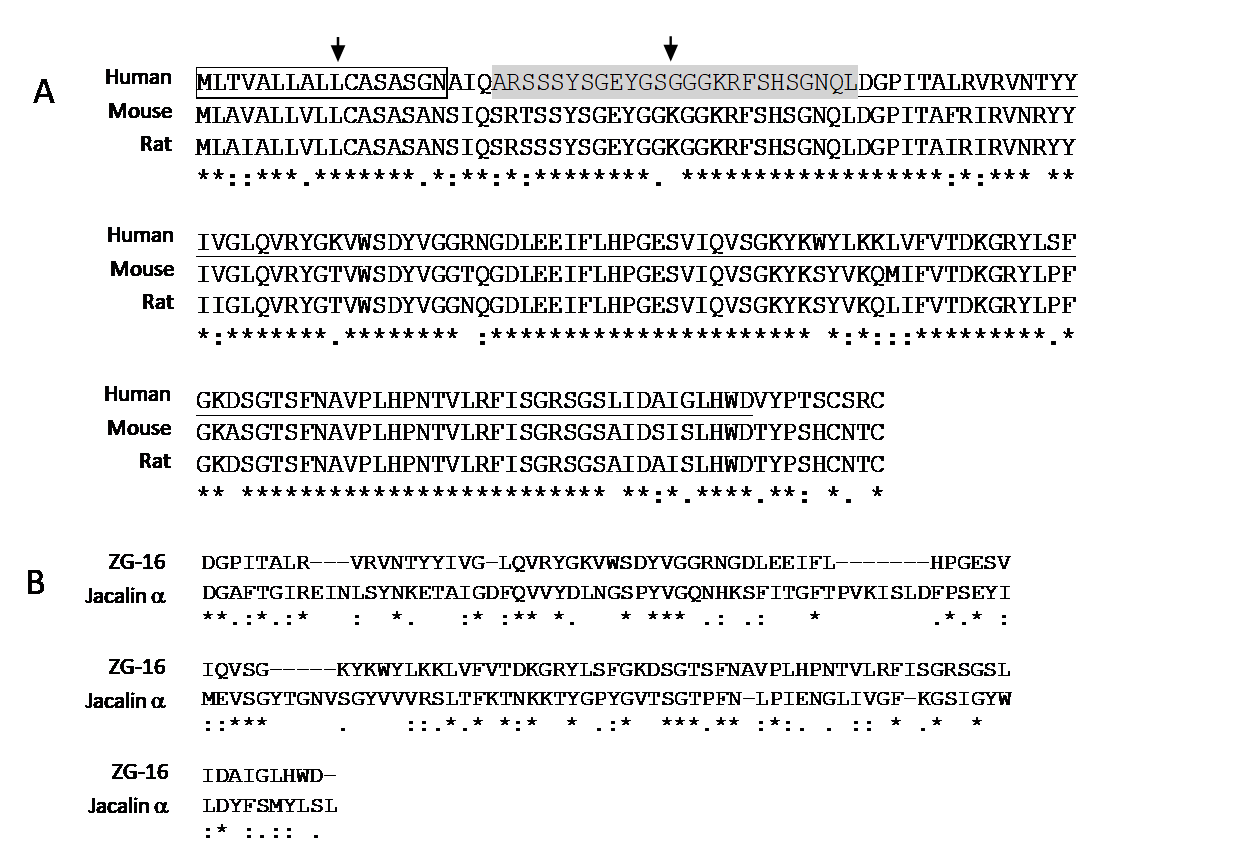


**Additional file 2: Figure S6. Protein sequence analysis of ZG16.** A: Sequence comparison shows 83% identity among the homologues of human, mouse and rat. Arrow with boxed sequence indicates signal peptide. Sequence highlighted in grey was synthesized for polyclonal antibody production. Sequence underlined is jacalin-like lectin domain. B. Human ZG16 has 28 % identity and 58% similarity with Jackfruit Jacalin α chain.

**Additional file 2: Figure S7. Subcellular localization prediction of ZG16.** We applied COMPARTMENTS (https://compartments.jensenlab.org) to predict subcellular localization of ZG16 protein. This analysis shows that the protein is more likely localized at Golgi lumen and extracellular matrix.
